# A Nutritional Approach to the Prevention of Cancer: from Assessment to Personalized Intervention

**Published:** 2016-01-31

**Authors:** L. Di Furia, M.R. Rusciano, L. Leonardini, P. Rossi, C. Giammarchi, E. Vittori, S. Tilocca, F.L. Russo, P. Montuori, M. Triassi, A. Nardone, M.D. Giaimo, M. Migazzi, S. Piffer, A. Iaria, A. Trapasso, A. Firenze, R. Cristaudo, M. Revello, A. Castiglion, V. Zagonel, G. Iaccarino, A. Addis, L. Natale, C. Di Somma, A. Colao, A. Perra, K. Giova, N. Montuori, M. Illario

**Affiliations:** 1 ARS Regione Marche, Federico II University, and R&D Unit, Federico II University Hospital; 2 Department of Translational Medical Sciences, Federico II University, and R&D Unit, Federico II University Hospital; 3 Regione del Veneto - Progetto Mattone Internazionale; 4 Ministero della Salute, Direzione generale della comunicazione e dei rapporti europei e internazionali; 5 ASL8 Regione Sardegna; 6 ASL Salerno regione Campania; 7 Dipartimento di Sanità Pubblica, Università Federico II; 8 Direzione salute e coesione sociale- Regione Umbria; 9 Servizio Epidemiologia Clinica e ValutativaAPSS – Trento; 10 Regione Calabria; 11 Università degli studi di Palermo Dipartimento di Scienze per la promozione della salute e materno Infantile (DSPSMI) “Giuseppe D’Alessandro”; 12 Azienda USL della Valle d’Aosta; 13 Regione Piemonte; 14 U.O.C. Oncologia Medica 1, Istituto Oncologico Veneto, IRCCS, Padova; 15 Dipartimento di Medicina e Chirurgia, Università di Salerno; 16 Agenzia sanitaria e sociale regionale-Assr Emilia-Romagna; 17 Dipartimento Salute e Risorse Naturali - Regione Campania; 18 IRCCS SDN Napoli, Universita’ Federico; 19 Dipartimento Di Medicina Clinica E Chirurgia, Sezione Di Endocrinologia, Universita’ Federico; 20 Istituto Superiore di Sanita, Roma; 21 ASL Napoli 3 Sud Regione Campania; 22 Reference Site Campania EIP on AHA-AOU Federico II UOS Ricerca e Sviluppo

**Keywords:** Nutritional interventions, cancer prevention, lifestyle and cancer risk

## Abstract

Among lifestyle factors, nutrition is one of the most important determinants of health, and represents a pivotal element of cancer risk. Nonetheless, epidemiological evidences of the relationship between several cancers and specific foods and nutrients is still inadequate, and solid conclusions are missing.

Indeed, caloric restriction without malnutrition is associated to cancer prevention. Food may be also the primary route of exposure to contaminants such as metals, persistent organic pollutants, and pesticides. Exposuredisease associations and the interplay with genetic susceptibility requires further studies on genetic variation, environment, lifestyle, and chronic disease in order to eliminate and reduce associated health risks, thus contributing to improve health outcomes for the population.

A primary nutritional approach for Active and Healthy Ageing (AHA) has been developed by the Nutrition group of the European Innovation Partnership (EIP) on AHA. The working group on lifestyles of the Italian Ministry of Health has developed a comprehensive approach to adequate nutrition using a consensus methodology to collect and integrate the available evidences from the literature and from the Italian experiences at the regional level, to raise the interest of other experts and relevant stakeholders to outline and scale-up joint strategies for a primary nutritional approach to cancer prevention.

## I. INTRODUCTION

Nutrition plays a key role in determining health outcomes of the population, and its impact depends upon the different stages of life, hence an effective approach for healthy nutrition should take into the account the needs of different target populations. Many foods and nutrients have long been studied for cancer prevention, and several macronutrients, micronutrients (vitamins and minerals), and non-nutrients (phytonutrients from plants, such as betacarotene) have been reported to contribute to cancer prevention. This review has been developed by a national panel of experts in cancer research, prevention, epidemiology, public health, and policy, and reflects an updated evidence related to dietary patterns and cancer risk.

## II. EPIDEMIOLOGY AN OVERVIEW ON EU, FOCUS ON ITALY

The availability of updated statistical data is very important for planning and evaluation of effective tumor prevention programs. In 2012 in Europe there were just over 3.4 million new cases of cancer (excluding nonmelanoma skin cancers), 53% (1.8 million) occurring in men and 47% (1.6 million) in women. The most common cancer sites were breast cancer (464,000 cases, 13.5% of all cancer cases), followed by colorectal cancer (447,000, 13.0%), prostate cancer (417,000, 12.1%) and lung cancer (410,000, 11.9%) (1).

The number of cancers in Italy was estimated for the year 2012: the most common cancer was colorectal, with over 54,000 new diagnoses in both sexes, followed for women by breast, prostate and lung cancers (50 396, 42 604 and 36 555 incident cases estimated, respectively). Incident cases for other types of cancer were significantly lower, whereas the prevalence data were higher (more than 10 times the incidence) for breast cancer, colon-rectum and prostate, but was significantly lower than other types of cancer, considered as a consequence of their poor prognosis (lung, stomach) or of their low incidence rate (melanoma, cervix). The highest mortality rates were related to lung cancer in men (80 per 100,000 person-years) and breast cancer in women (35 per 100,000 person-years) and the lowest for melanoma in both sexes, and for cervical cancer in women. For colorectal, lung, stomach cancer and melanoma, all indicators were higher in men than women, with the exception of the prevalence of melanoma (2).

## III. RELATION BETWEEN LIFESTYLE AND CANCER RISK

Only 5–10% of all cancers seems to be attributed to genetic defects, whereas the remaining over 90% depends upon environment and lifestyles, which include smoking, diet (fried foods, red meat), alcohol intake, sun exposure, environmental pollutants, infections, stress, obesity and physical inactivity.

**Figure 1.**
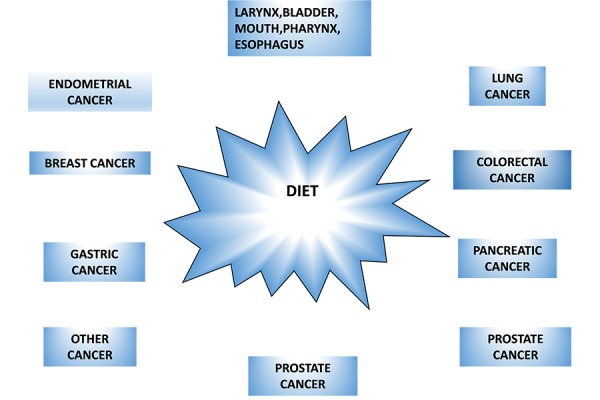
Relationship between food intake and cancer.

A number of evidences indicate that the main cause for all cancer deaths is related to the diet, (Tab 1), that makes nutrition the leading cause of cancer (Figure 1).

**Figure 2.**
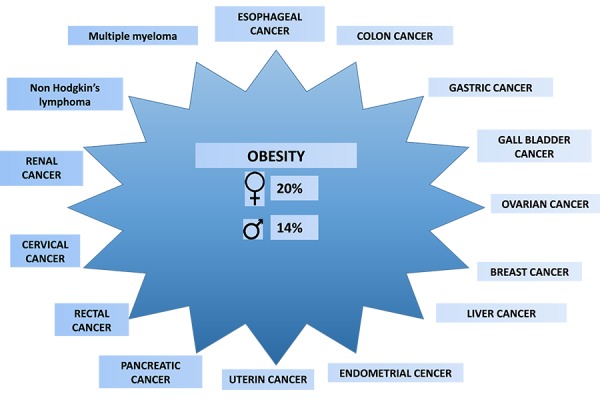
Obesity and cancer mortality.

The primary hypothesis of the present paper is that cancer prevention requires a complex approach including nutritional and behavioral interventions. Indeed, the impact of nutrition and obesity is now demonstrated both in cancer incidence and in cancer mortality (3) (Figure 2). Moreover, since 18% of incident cancers occurs in people who have had a tumor, it is important to educate these people to a style of life (in particular nutrition and physical activity) suitable for reducing the risk of a second cancer (4).

In recent years a growing number of studies and several meta-analyses have also shown that physical activity is associated with decreased risk of relapse in patients with breast cancer and colorectal cancer (5). For this evidences, the American Society of Medical Oncology published guidelines in which at least moderate physical activity is recommended in patients long surviving from cancer to reduce the risk of cancer relapse (6). Prevention of obesity-associated comorbidities (particularly diabetes, cardiovascular disease and hypertension), must be a priority objective in the long surviving patients (7). These diseases not only in fact contribute in determining the outcome of cancer care (reducing toxicity related to treatment), but also increase the probability of relapse and death rates cancer-specific and not cancer-specific (8–10).

The most important European prospective cohort study on cancer and nutrition (EPIC: The European Prospective Investigation into Cancer and Nutrition) involves more than half a million participants recruited in 10 European countries, who have been monitored for almost fifteen years. EPIC studied the correlation between diet, nutritional factors, anthropometry, physical activity and cancer risk, and has been providing a significant contribution to the understanding of the effects of specific factors on the risk of cancer (11–16), In particular, in the context of the EPIC project a prospective multicenter study was carried out (17) in 23 countries, including Italy, which describes of the existence of some associations between specific foods and risk of occurrence of certain cancers in a very large population.

Another important aspect of nutrition and cancer incidence is related to environmental pollution from illegal waste disposal. These data are pivotal to support the development of appropriate public health strategies, and to identify and carry out prevention activities to reduce the overall risk of cancer. According to WHO contaminated sites are “areas hosting or having hosted human activities which have produced or might produce environmental contamination of soil, surface or groundwater, air, food-chain, resulting or being able to result in human health impacts” (18, 19). Protecting human health from exposure to environmental pollutants is a public health priority, but the burden of tumors among residents in areas contaminated by carcinogens is not adequately studied and the specific epidemiological literature on cancer risk in polluted areas is limited. In Italy, SENTIERI Project (Epidemiological study of residents in National Priority Contaminated Sites, NPCSs), funded by the Italian Ministry of Health, studied mortality among residents of 44 NPCSs included in the “National Environmental Remediation Programme” (20, 21). In both genders an excess was observed for overall cancer incidence (9% in men and 7% in women) as well as for specific cancer sites (colon and rectum, liver, gallbladder, pancreas, lung, skin melanoma, bladder and Non Hodgkin lymphoma). Deficits were observed for gastric cancer in both genders, chronic lymphoid leukemia (men), malignant thyroid neoplasms, corpus uteri and connective and soft-tissue tumors and sarcomas (women) (22). An interesting review on environmental pollution and health effect was recently published by Triassi et al (23). This review analyzes all evidences from published data concerning cancer, childhood mortality, birth defects and human biomonitoring, despite the methodological and sampling heterogeneity between studies, in order to establish the current situation, knowledge gaps and research priorities. Although many intrinsic limits affect contrasting studies and data (24, 25), overall available findings point out a possible long term role of waste, as suggested by positive correlation with outcomes such as liver and lung cancer mortality, in addition to a wasterelated short term effect (less than 1 year). The latter was confirmed by association with congenital malformation, which is compatible with the lack of remediation of the polluted sites and persistence of waste mismanagement to date. Research on exposure to pollutants confirms a possible exposure to illegal waste fires in Campania “triangle of death”, nevertheless no risk excess for diseases has been recognized to be related to incineration (sarcomas, non-Hodgkin lymphomas), and has been detected in resident population. Further studies are needed to better define waste related-health effects. However, this review compiles the data in a harmonized and effective way, so that the current status, knowledge gaps and research priorities can be established by further studies pivotal to support the development of appropriate public health strategies, and to identify and carry out prevention activities aimed at reducing the overall risk of cancer.

## IV. ANALYSIS OF THE RELATION AND MECHANISMS UNDERLYING THE EFFECTS OF NUTRITION ON CANCER

Food is an important factor in determining cancer incidence in many countries and regions. It can have both positive (carcinogenic) and negative (preventive) effects. An excessive intake of food is one of the main factors of neoplastic risk and it is proved that obesity is a condition that predisposes the development of malignant neoplasm. Overweight is responsible for 14% of cancer deaths in men and 20% in women. Recent studies say that a diet rich in selenium and Omega-3 has a preventive role in prostate carcinoma, while a diet rich in animal fats is responsible for increase of incidence in breast cancer (BC). Retinoids and vitamins reduce the risk of BC in women with a body mass index (BMI) > 25 Kg/mq; they have a preventive action on both gastric cancer caused by Helicobacter Pilory and hepatocarcinoma caused by hepatitis B and C (3–11).

### IV.1 Red meat and colon cancer

Bingham et al (26), have shown the important relationship between red meat and colon-rectal cancer; the heterocyclic amines produced in cooked meat are related to breast-cancer.

### IV.2 High-fat diet and breast cancer

Published data from epidemiological and case-control studies on the association between high fat intake and BC risk have been conflicting and may be attributable to difficulties in obtaining accurate information on fat intake as well as because of limited heterogeneity of intake within a specific geographic area from which the study cohorts come.. The study conducted by Sieri (27) reported that high total and saturated fat intake were associated with greater risk of ER+/PR+ BC. High saturated fat intake was also associated with greater risk of HER2 negative disease.

### IV.3 Vitamins e and prostate cancer

Studies done in the 1980s and 1990s suggested that vitamin E and selenium each somehow provided protection against prostate cancer (PC). In 2001, the US National Cancer Institute initiated the Selenium and Vitamin E Cancer Prevention Trial (SELECT), which tested whether selenium (Se; 200 μg/d from Lselenomethionine), vitamin E (400 IU/d of all rac-α- tocopheryl acetate) or both could reduce PC risk (28). Study supplementation stopped 3 years before the expected trial end date because interim analyses showed very low likelihood of benefit with continued intervention (29). At that time, vitamin E alone modestly increased PC risk (hazard ratio [HR] = 1.13; P < .06); with additional follow-up, this became statistically significant (HR = 1.17; P < .008) (30).

Recently, a new report in the Journal of the National Cancer Institute clarifies the picture (31). This study showed that to enail selenium, in the absence of supplementation, was not associated with PC incremental risk. Selenium supplementation (combined selenium only and selenium + vitamin E arms) had no effect among men with low selenium status (<60th percentile of toenail selenium) but increased the risk of high-grade PC among men with higher selenium status by 91% (P = .007).

### IV.4 Vitamin D and colon cancer

A recent large observational study (32) investigated the association between circulating levels of vit. D (25-(OH)D), dietary intake of calcium and vitamin D, and the risk of colorectal cancer in European populations. The results of the study demonstrated the existence of a strong inverse correlation between the concentration of 25-(OH) D and the risk of cancer of the colon and rectum. Compared to an average concentration of default level of 25- (OH)D (50.0 to 75.0 nmol/l), lower levels were associated with an increased risk of colorectal cancer (<25.0 nmol/l) whereas the highest concentrations associated with lower risk (75.0 to 99.9 nmol/l). Even, a greater dietary intake of calcium was associated with a lower risk of colorectal cancer. Further randomized clinical trials are needed to assess whether the increased circulating concentrations of 25-(OH)D can effectively reduce the risk of cancer of the colon and rectum.

### IV.5 Vitamin B and lung cancer

B vitamins, including vitamin B6 and folate (B9), are essential for DNA synthesis and methylation.. Until now, studies on B vitamins focused on the correlation with colorectal cancer, but did not generate convincing results (33,34). The The EPIC study, however, investigated whether the factors involved in the metabolism of ‘1- carbon were associated with the onset of lung cancer, by a comprehensive investigation on serum B vitamins and methionine. Case and control study participants quartile were compared to serum levels of each of the four B vitamins as well as to homocysteine and methionine. A significantly lower risk of lung cancer was observed in subjects with a higher intake levels of vit. B6, as well as for higher levels of methionine (35).

### IV.6 Resveratrol and cancer

Resveratrol (3,5,4′-trihydroxy-trans-stilbene) is a natural antioxidant found largely in the skins of red grapes, which are used to make wines, with proved benefits on metabolism, heart disease and cancer. Studies made on animal models revealed that resveratrol could have positive effects on breast- colorectal- liver- pancreas and prostate-cancer, but it depends upon delivery system, timing, dosage and experimental models used. Although the molecular targets of resveratrol have been identified, less is known about the mechanism of its antitumoral activity, although it has been recognized that resveratrol influences different transduction pathway involved in cancer onset (36).

## V. MECHANISMS UNDERLYING THE EFFECTS OF NUTRITION ON CANCER RISK

Nutrients affect gene expression by epigenetic mechanisms, that are potentially reversible, heritable changes in gene expression that do not modify the DNA sequence. The main mechanisms of epigenetic control in mammals are:

- DNA methylation,
- Histone modifications,
- RNA silencing.

The potential reversibility of epigenetic changes suggests that they can be modulated by nutrition and bioactive compounds in foods, and may mediate environmental signals, thus connecting the genes of tumor susceptibility to environmental factors in the etiology of cancer (37). Indeed, different bioactive food components (BFCS) with anticancer potential, including folic acid, polyphenols, selenium, retinoids, fatty acids, isothiocyanates and allyl compounds, can regulate DNA methylation and histone modification processes. These activities influence the expression of genes involved in proliferation, differentiation and cell death that are the biological phenomena most frequently altered in cancer. Although epigenetic alterations represent a promising target for cancer prevention with BFCS, few studies have addressed the effects of dietary components on these mechanisms in vivo. Furthermore, diet–epigenome interactions also occur in utero, the impact of nutrition on the early stages of life compared to the risk of developing tumors should be further investigated (38). An example of the link between cancer and nutrition is offered by the fat-rich diet. Some soluble factors secreted by adipocytes and macrophages, such as TNF-alpha and other inflammatory proteins, link inflammation and tumors. Many cytokines, such as IL-1, IL-6, IL-8, IL-32, IL-33 and MCP-1, are biomarkers of tumors and of certain chronic diseases, and are linked to the transcription factors NFκB and AP1, that control genes regulating cell metabolism and biological functions such as differentiation and cell proliferation (39).

## VI. SCREENING AND ASSESSMENT TOOLS

To date, a number of tools have been developed, tested and validated for the assessment of these domains. A thorough overview has been provided by Bousquet et al (40), although comprehensive tools to evaluate the nutritional state are still missing.

Validated tools for the assessment of nutritional state would be useful for the stratification of the population, as well as for the identification of innovative biomarkers (for osteopenia, sarcopenia etc) that would provide a personalized approach to improve the health condition and outcomes of interventions.

Screening assessment should take into the account:

- To educate and coaching people about the benefits of a balanced diet, and cancer risk linked to poor dietary habits
- Healthy lifestyle, in order to provide a correct dietary approach for wellbeing
- Psycosocial behavior evaluation that could contribute to inadequate energy intake
- Identification of innovative biomarkers concerning inflammatory process, lipidic and glycemic metabolism for personalized nutritional interventions.

Large scale validation across different EU countries would provide evidences to ground targeted and sustainable interventions to carry out in the different loco-regional and socio-cultural contexts.

## VII. CURRENT NUTRITIONAL APPROACH FOR CANCER PREVENTION: THE ITALIAN EXPERIENCE

A primary nutritional approach for Active and Healthy Ageing (AHA) has been developed by the Nutrition group of the European Innovation Partnership (EIP) on AHA that could prove effective also to sustainable cancer prevention strategies.

The nutritional approach for cancer prevention might take advantage from the model developed by the Nutrition group of the EIP on AHA. The group developed a common vision on an integrated nutritional approach as a sustainable tool to prevent malnutrition in older people and promote active and healthy ageing (42) and on a sustainable approach to malnutrition based upon the integration of assessment and stratified interventions.

That approach is shared by the working group on lifestyle of the Italian Ministry of Health (MoH). Italian MoH has been supporting several nutritional interventions to promote a correct lifestyle in order to prevent cancer. Such interventions are reported into the website of Italian MoH (www.salute.gov.it). Thanks to policy documents (GainHealth) and National Plans (National Plan of Prevention), Italy has strengthened its actions to promote a healthy lifestyle, developing an “inter-sectoral” and “crosssectoral” approach to influence the life style choices against unbalanced eating habits, physical inactivity, smoking, alcohol abuse and to create the conditions that promote a correct environmental context (redefinition of urban planning to favor the path on foot or the use of cycling, improving the availability of healthy eating options, air quality, guarantee healthy and safe environments in school and workplaces etc.) (see also http://www.quadernidellasalute.it/download/download/24-luglio-dicembre-2014-quaderno.pdf).

Among the national intervention aimed to promote a correct lifestyle for preventing cancer, the most successful are:

- “**Okkio alla Salute** (http://www.epicentro.iss.it/okkioallasalute/) that is aimed to a surveillance system on overweight and obesity in primary school children (6–10 years) and on the related risk factors. The main objective is to describe the geographical variation and evolution over time of weight, eating habits, levels of physical activity in children, and identifies school activities favoring healthy nutrition and exercise, in order to implement initiatives for the improvement of living conditions and health of children in primary schools. ”Okkio alla Salute” started in 2007 in the context of another project, “System of surveys on behavioral risks in age 6–17 years”, that was promoted and financed by the Ministry of Health/CCM, and is coordinated by the National Center for Epidemiology, Surveillance and Health Promotion (CNESPS) Institute of Health (ISS) in collaboration with the Regions, the Ministry of Health and the Ministry of Education, University and Research. “Okkio alla Salute” is connected to the European program “Gaining health” as well as to the national and regional prevention plans, and is part of the European Region of the World Health “Childhood Obesity Surveillance Initiative (COSI)”.
- **The HBSC** (Health Behaviour in School-aged Children - Health-related behaviors in children of school age) project, that is a multicenter international study (www.hbsc.org) in collaboration with the Regional Office of the World Health Organization to Europe (www.who.int/about/regions/euro/en/index.html). The main objective is to increase the understanding about adolescent health and well-being, and to use the results obtained by the survey to guide the practices of health promotion and youth policies both nationally and internationally.
- **PASSI project**. This national surveillance project of public health that collects, through continuous and through surveys, information on the Italian adult population (18–69 years) on lifestyle and behavioral risk factors associated with the onset of chronic noncommunicable diseases and the degree of knowledge and adherence to the intervention programs that the country is building for their prevention. PASSI monitors the achievement of health goals set by the national and regional health plans, and contributes to the assessment of the National Prevention Plan because knowing the profiles of health and risk factors of the population is a prerequisite for implementing prevention activities specifically targeted to vulnerable groups, required to monitor and evaluate the effectiveness of the interventions. PASSI is designed as a surveillance system run by the local health authorities, and adapted to the different locoregional contexts, that is therefore constructed as a three levels system:
  - a corporate level with the activities of detection, data logging, analysis and communication to local communities
  - a level of regional coordination, which also provides analysis and reporting to regional planners
  - a central level responsible for planning, research, training and development.

## VIII. MEDITERRANEAN DIET

Many initiatives focus on the adherence to the Mediterranean Diet (MD) as a model for a correct lifestyle approach to nutrition. It exemplifies the concept of local food production chain, food safety and adequate nutritional interventions through a primary approach. Adherence to Mediterranean diet (MD) is reportedly declining in the last decades. Highest prevalence of adherence to MD was observed during the years 2005– 2006 (31.3%) while the prevalence dramatically fell down in the years 2007–2010 (18.3%; P<0.0001). The decrease was stronger in the elderly, less affluent groups, and among those living in urban areas (42). Several studies recommend the Mediterranean diet and daily physical activity to prevent cancer development. These recommendations, however must be transformed into public health structured programs, so that they assume operational effectiveness. The aim of the diet and lifestyle intervention called Med-Food Anticancer Program (MFAP) is to promote the Mediterranean diet and physical activity in the adult population. In particular; the target for participants in the intervention is the increased consumption of legumes, fish, whole grain bread and cereals, fruits and vegetables, and the decreased consumption of meat, cheese and foods of animal origin. At the same time, it is recommended to make at least ten thousand steps a day (43). A primary nutritional approach, like the case of the Mediterranean Diet, takes advantage from culinary interventions, that can sustainably support healthy choices and takes also into account the entirety of needs that influence the adequacy of nutritional intake. A sustainable approach integrates culinary and clinical interventions aiming to:

- Strengthen the general awareness and knowledge of the effects of nutrition on health in general, and on the risk of cancer in particular
- Develop an integrated approach for nutritional interventions during the entire life course by taking actions on different age groups of the population
- Extrapolate the transferable and contextual elements from the MD primary approach to healthy nutrition and lifestyle.

The challenge that lies ahead for multidisciplinary teams, involving clinicians, nutritionists, dietitians and chefs is to scale-up this model and adopt it to other regional context, taking advantage from local traditions and dietary habits.

## IX. OTHER NUTRITIONAL APPROACHES FOR CANCER PREVENTION

Availability of food ingredients greatly varies across EU loco-regional contexts. The availability of food supplements, vitamins and minerals are relevant to good health for which it is necessary to provide a full range in sufficient quantities through the diet. Nutraceuticals and functional foods, bioactive molecules and innovative products with added value, capable of incorporating bioactive components (antioxidants, pigments, polysaccharides, proteins, peptides, protein hydrolysates, amino acids, fatty acids, lipids), are currently available, which have been identified in healthy foods of the Mediterranean diet (obtained from industries of tomato, wine, milk, olive oil and citrus), or of marine origin. Nutritional interventions estimates will benefit from the production of innovative functional foods (such as dairy products fortified), properly tested on specific target populations.

Functional foods (Regulation EC 178/2002) and fortified are important to ensure sustainable dietary health. They can be considered as carriers of functional ingredients with specific health benefits, but are subjected to health claims regulation (EC 1924/2006), which requires the information provided to consumers on the label to be based on scientific evidence, to prevent consumers from being misled by unclear information, or incorrect, and false claims. A great contribution in this field was given by the recent Regulation (EU) No 432/2012 of 16 May 2012, establishing a list of permitted health claims made on foods, other than those referring to the reduction of disease risk, to children’s development and health. One of the objectives of both Regulations (EC) No 1924/2006 and (EU) No 432/2012, is to ensure that health claims are truthful, clear, reliable and useful to consumers. In the area of health claims, the study of beneficial microbes has been approved in the European Union, which is a way for the yogurt to improve lactose tolerance. Research conducted in thirteen EU countries plus Switzerland (35) revealed that five EU Member States have already adopted national nutritional guidelines or recommendations that include both probiotics or fermented milk with live bacteria, recognizing the health benefits associated with consumption of live microbes, although commercial marketing is not authorized yet. Selective use of functional products is linked not only to the lack of awareness about the health benefits due to their consumption, but also to the absence of a clear link between a specific condition and its “treatment”/prevention through the consumption of specific functional products. In the light of the above considerations, it would be important to: i) integrate recommendations and indications approved for health functional foods, that could benefit all consumers and public health; ii) create nutritional guidelines, including old and new functional products, highly specific for disease prevention or integration with medical prescriptions.

## X. DISSEMINATION

The promotion of healthy nutrition should be carried out adequately targeting the different age groups, also taking advantage of high visibility and popular events, stimulating the adoption of a balanced and varied diet, and of an active lifestyle. The use of dietary supplements should be appropriate to respond to specific health needs. Educational opportunities targeted to specific categories of stakeholders (such as school networks hotel, dieticians, clinicians, pharmacists, general practitioners, nurses, chefs, informal carers, voluntary organizations) should involve them in the development of targeted programs to carry out an approach directed to the entire lifespan, to improve the nutritional awareness of each age group. Formal training programs, such as training for chefs, PhDs, masters and specializations should be coherently developed within this integrated nutritional approach.

All stakeholders involved in the follow-up of cancer patients should educate long-surviving patients to remain vigilant, and favor the access to programs of primary and secondary prevention, helping to maintain a healthy lifestyle to reduce the risk of relapse and second cancers.

## XI. INNOVATIVE SOLUTIONS: ICT TOOLS

A stepwise approach to malnutrition would close gaps of knowledge by a common structured approach to stepwise adequate interventions (primary/secondary/tertiary), based on a unified assessment and modular ICT solutions. Assembling all the validated screening, assessment and monitoring tools on nutrition in an ICT-supported pyramid interrelated to the action pyramid, like the one proposed by the EIP on AHA Nutrition Action Group: Nutrilive (41), could prove effective also to sustainable cancer prevention strategies.

The Nutrilive service infrastructure would support these services and reach users efficiently, while also offering an ICT platform for shared data management.

Nutrilive would:

- provide an interoperable infrastructure to support different types of services, including screenings and self-assessment
- allow the processing of data from heterogeneous sources
- provide support information to different users
- be interoperable with other existing information systems.

The Nutrilive platform will also allow to collect recipes and menus that represent a significant part of the cultural heritage of our country: in fact, some eating habits are developed over many years and have been handed down in the oral tradition from one generation to the next. Collection of recipes, menus and stories related to their tradition would bring added value and would support the cultural exchange.

## XII. CONCLUSIONS

A comprehensive approach to adequate nutrition is very important for cancer prevention. The Italian group on lifestyle of the Italian Ministry of Health shares the approach of the Nutrition group of the EIP on AHA, that developed a common structured approach to stepwise adequate interventions (primary/secondary/tertiary), based on a unified assessment and modular ICT solutions. Such strategy could prove effective also to sustainable cancer prevention strategies conjugating surveillance, screening and self assessment programs, with data collection and analysis, offered through a substrate for shared data management.

## Figures and Tables

**Table 1.** **Percentage of cancer deaths related to diet**

TYPE OF CANCER	% OF CANCER DEATHS
LARYNX, BLADDER, MOUTH,PHARYNX, ESOPHAGUS	20
LUNG CANCER	20
COLORECTAL CANCER	70
PANCREATIC CANCER	50
PROSTATE CANCER	75
PROSTATE CANCER	50
OTHER CANCER	10
GASTRIC CANCER	35
BREAST CANCER	50
ENDOMETRIAL CANCER	50
